# Ratio of early transmitral inflow velocity to early diastolic strain rate predicts atrial fibrillation following acute myocardial infarction

**DOI:** 10.1007/s10554-023-02991-y

**Published:** 2023-11-13

**Authors:** Caroline Løkke Bjerregaard, Flemming Javier Olsen, Mats Christian Højbjerg Lassen, Anne-Sophie Winther Svartstein, Thomas Fritz Hansen, Søren Galatius, Allan Iversen, Sune Pedersen, Tor Biering-Sørensen

**Affiliations:** 1 Department of Cardiology, Copenhagen University Hospital - Herlev and Gentofte, Hellerup, Denmark; 2 Department of Biomedical Sciences, University of Copenhagen, Copenhagen, Denmark; 3 Department of Cardiology, Copenhagen University Hospital – Bispebjerg and Frederiksberg, Copenhagen, Denmark; 4 Cardiovascular Non-Invasive Imaging Research Laboratory, Department of Cardiology, Copenhagen University Hospital - Herlev and Gentofte, Gentofte Hospitalsvej 8, 2900 Hellerup, Denmark

**Keywords:** STEMI, Diastolic function, Atrial fibrillation, Strain imaging

## Abstract

The ratio of early transmitral filling velocity to early diastolic strain rate (E/SRe) has been proposed as a new non-invasive measurement of left ventricular filling pressure. We aimed to investigate the ability of E/SRe to predict atrial fibrillation (AF) after ST-elevation myocardial infarction (STEMI). This was a prospective cohort study of patients (n = 369) with STEMI. Patients underwent an echocardiographic examination a median of two days after pPCI. By echocardiography, transmitral early filling velocity (E) was measured by pulsed-wave Doppler, and early diastolic strain rate (SRe) was measured by speckle tracking of the left ventricle. E was indexed to SRe and the early myocardial relaxation velocity (e’) to obtain the E/SRe and E/e’, respectively. The endpoint was new-onset AF. During follow-up (median 5.6 years, IQR: 5.0–6.1 years), 23 (6%) of the 369 patients developed AF. In unadjusted analyses, both E/SRe and E/e’ were significantly associated with AF [E/SRe: HR = 1.06; (1.03–1.10); p < 0.001, per 10 increase] and [E/e’: HR = 1.11 (1.05–1.17); p < 0.001, per 1 increase] and had equal Harrell’s C-statistic of 0.71. However, only E/SRe remained an independent predictor after multivariable adjustments for clinical and echocardiographic parameters [E/SRe: HR = 1.06 (1.00–1.11); p = 0.044, per 10 increase]. E/SRe was further significantly associated with AF in patients with E/e’ < 14 HR = 1.09 (1.01–1.17); p = 0.030, per 10 increase), also after multivariable adjustments. E/SRe is an independent predictor of AF in STEMI patients, even in subjects with seemingly normal filling pressure.

**Supplementary Information:**

The online version contains supplementary material available at 10.1007/s10554-023-02991-y.

## Introduction

Atrial fibrillation (AF) is the most common cardiac arrhythmia of clinical significance [1]. Worldwide age-adjusting prevalence of AF, was estimated in the 2017 Global Burden of Disease Study to be approximately 37.6 million people [2]. The chance of developing AF increases with advancing age and due to the higher aging of the population, the incidence of AF will approximately double with each decade of advanced aging above 60 years [3], and the incidence is projected to grow at least three-fold by 2050 [4]. This number might be even greater since many patients with AF remain undiagnosed [5, 6]. AF is related to an increased risk of stroke, peripheral embolism, and mortality [7, 8].

The common approach for detecting AF currently relies on intermittent, symptom-based, long-term recordings or Holter monitoring all with limited diagnostic capabilities [9]. Monitoring the heart rhythm continuously for 3 years with cardiac monitors using an AF-specific algorithm has previously revealed that 27% of acute myocardial infarction (AMI) patients with left ventricular (LV) dysfunction develop AF [10]. Furthermore, patients with AF events > 30 s face an elevated risk of thromboembolic complications. Consequently, there is a need for establishing markers that could indicate a high risk of AF to determine which patients could benefit from long-term rhythm monitoring [11]. Echocardiography is routinely performed after AMI, and echocardiographic measures of LV filling pressure could be useful risk markers for AF.

The tissue Doppler-based measure E/e’ is traditionally used as a measure of LV filling pressure and is a key measure to evaluate diastolic dysfunction following AMI [10, 12]. However, the E/e’ has several technical limitations including susceptibility to sampling location and angle dependency that challenges its interpretation, particularly after AMI [13]. Myocardial speckle tracking allows for the acquisition of the early diastolic strain rate, a measure of LV relaxation similar to e’. However, as opposed to e’, early diastolic strain rate is measured from all LV segments and is angle independent. By indexing this to early transmitral inflow velocity, a novel index of LV filling pressure is acquired; the E/SRe, which has shown to be correlated with invasively measured LV filling pressure [13, 14].

While the association between E/e’ and AF is well-established, studies have not shown consisting results regarding the association between E/SRe and AF [15, 16]. We sought to investigate the ability of E/SRe as a predictor of AF after AMI.

## Methods

### Population

This was a prospective study of acute ST-segment elevation myocardial infarction (STEMI) patients treated with primary percutaneous coronary intervention (pPCI) at Gentofte Hospital, Denmark during the period of September 2006 to December 2008. A total of 391 patients were included and underwent a detailed echocardiographic examination. Four patients were excluded due to inadequate quality of the echocardiographic examination, 14 due to known AF, and 4 due to missing transmitral Doppler flow. This left 369 patients eligible for analysis in this study. The study population has previously been described in detail [17–19].

### Clinical data

Baseline data including demographic and clinical parameters relevant for this study was obtained prospectively when the patients were enrolled in the study. Hypertension was defined as the use of blood pressure-lowering drugs on admission. Diabetes mellitus was defined as non-fasting plasma glucose concentration ≥ 11.1 mmol/L, fasting plasma glucose concentration ≥ 7 mmol/L, or the use of glucose-lowering drugs at admission. Troponin I was measured immediately upon admission.

### Echocardiography

Echocardiography was performed using Vivid 7 ultrasound system (GE Healthcare, Horten Norway) using a 3.5-MHz transducer by trained sonographers. The echocardiographic examinations were performed within 5 days (median 2 days (IQR: 1–3)) after pPCI [20, 21]. All participants were examined with conventional two-dimensional echocardiography, pulsed-wave Doppler, and color TDI according to standardized protocols. The echocardiography was then stored and analyzed offline using commercially available software (EchoPAC, GE Healthcare, Horten Norway) by a single investigator who was blinded to the patients’ clinical characteristics and outcomes.

### Conventional echocardiography

Chamber quantifications were performed according to guidelines [22]. Left ventricular mass index (LVMI) was calculated by left ventricular mass divided by body surface area. LV ejection fraction (LVEF) was measured using the Simpson’s biplane method. Left atrial (LA) end-systolic volume was measured using the biplane area-length method in the apical 2- or 4- chamber view at end-systole. Two-dimensional parasternal long axis images were used to measure LV internal dimension at end-diastole (LVIDd) and LV end-systolic volume and LV end-diastolic volume were measured in the 2- and 4- chamber view at end systole and end diastole respectively. Peak velocities of transmitral early filling (E) and late filling (A) along with E/A-ratio and deceleration time (DT) of the E-wave were assessed using pulsed-wave Doppler in the apical 4- chamber view. Pulsed-wave tissue Doppler was used to measure peak longitudinal early diastolic tissue velocity (e’) with sample volumes placed at the lateral and septal mitral annular level in the apical 4-chamber view. The E/e’ was calculated from these Doppler measurements, and a high E/e’ was defined as above 14.

### Speckle tracking echocardiography

**Fig. 1:**
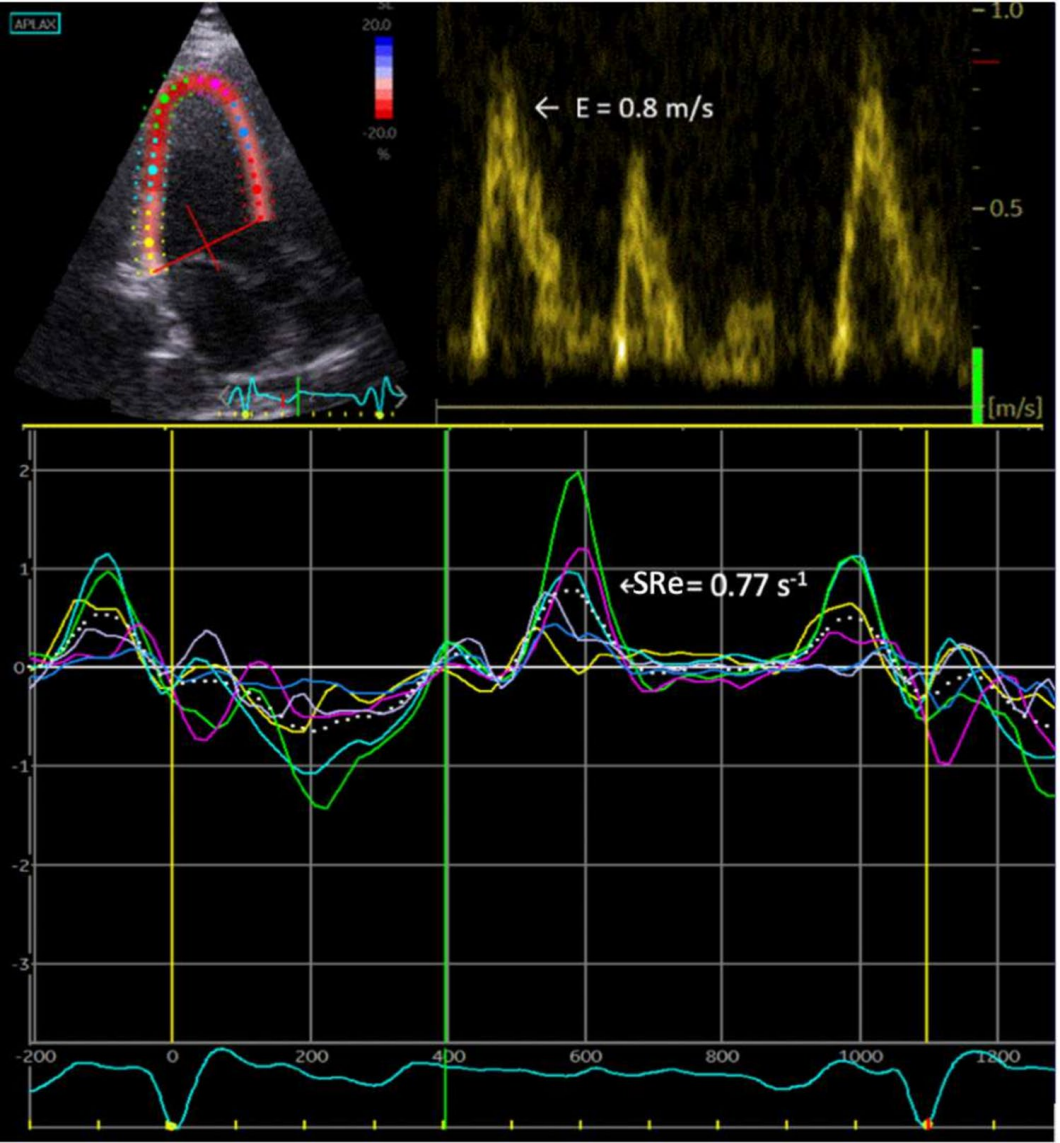
Example of E/SRe measurements**.** The figure shows an example of the measurements of early filling velocity (E) using pulsed-wave Doppler (top right), and early diastolic strain rate (SRe) from 2-dimensional speckle tracking analysis (top left and bottom). The white dotted line shows the global value

2D speckle tracking was performed in the 3 apical projections (4-, 2- and 3-chamber view) with the highest available frame rate (mean 86 ± 23 Hz). The region of interest (ROI) was defined by a semiautomated function and could be manually adjusted to avoid inaccurate tracking of the myocardial wall. A total of 18 segments, 6 per projection, were included. The global value was calculated as a mean of all LV segments. Segments were excluded if deemed untraceable due to shadow/artefacts. Global longitudinal stain (GLS) was calculated as the mean peak systolic longitudinal strain of all LV segments. The global SRe was calculated from the mean values of all LV segments. E/SRE was defined as E velocity divided by global SRe (Fig. 1). A total of 368 patients had 4-chamber views adequate for speckle tracking analysis, 362 subjects had adequate 2-chamber views and 354 had adequate 3-chamber view images for speckle tracking analysis. LA speckle tracking was performed in the apical 4-chamber and 2-chamber view by tracing the endocardial border manually using LV dedicated strain software to acquire peak atrial longitudinal strain.

The feasibility of E/SRe was 99% for the study population as we could not measure E/SRe in 4 of 373 patients. Previously our lab has shown good intra-variability and interobserver variability of both SRe with a small bias (for SRe: mean difference ± 1.96 SDs was 4 ± 23 m for the intraobserver analysis and 6 ± 37 m for the interobserver analysis [23].

### Outcome

The endpoint was the development of new-onset AF. The endpoint was obtained by International Classification of Disease (ICD-10) codes from the Danish Board of Health's National Patient Registry in 2013. The competing endpoint of all-cause death was obtained from the National Causes of Death Registry. Follow-up was 100%. The ICD-10 codes were obtained from all hospital contacts, both in- and outpatient contact for all the patients.

### Statistics

Normal distribution for continuous variables was assessed by histograms and box plots. Continuous variables showing normal distribution are presented as mean±standard deviation, whereas those not showing normal distribution are presented as median with interquartile ranges (IQR). Categorical variables are presented as total numbers and percentages. Groupwise comparison was made with the Student’s T-test for normally distributed continuous variables, and with the rank-sum test for variables not showing normal distribution. Categorical variables were compared with the Chi^2^-test. Comparisons were made according to the endpoint of AF and according to high vs. low E/SRe (defined by the median of 97 and confirmed by ROC curves as the optimal cut-off value with the highest AUC). Of note, among healthy participants from the Copenhagen City Heart Study, 14 of 1623 (0.9%) have an E/SRe above this threshold. Univariable proportional hazards Cox regression was used to investigate the association between both E/SRe and E/e’ and AF. Harrell’s C-statistics were calculated from the univariable regressions. Multivariable Cox regressions were made to adjust for confounders and obtain adjusted hazard ratios. Adjustments were made for: age, sex, hypertension, diabetes mellitus, peak Troponin I, LVMI, LVEF, heart rate, significant valve disease and left anterior descending culprit lesion. The multivariable model was extended to include LA strain in the subgroup of patients with feasible LA speckle tracking (n: 299, events: 18). Proportional hazards were assessed by Schoenfeld residuals. To avoid a potential issue of collinearity, E/SRe and E/e’ were investigated in separate multivariable models adjusted for the same confounders but were not included in the same model. Linearity was assessed by linear and quadratic effects. Test for interaction was made with sex and GLS as these have previously shown to modify the association between E/SRe and outcome. To investigate whether LVEF impacted the association between E/LVSRe and AF a test for interaction against LVEF was applied the association between E/SRe and the outcomes were depicted using restricted cubic splines based on a Poisson model, with the number of knots based on the lowest Akaike information criterion.

Cox regression was also applied in a subgroup analysis of patients with normal filling pressure defined as an E/e’ < 14. Competing risk regression analyses were performed with similar multivariable adjustments as noted above by the Fine and Gray method to account for death as a competing event (number of competing events: 52). Estimates are presented as subdistributional hazard ratios (SHR). Kaplan–Meier curves were created to show the association between high vs. low E/SRe and the combined outcome throughout the follow-up period. Statistical difference was tested with the log-rank test. All statistical analyses were performed with STATA Statistics/Data analysis, SE 15.1 (StataCorp, Texas, USA). A p-value < 0.05 was considered significant in all tests.

## Results

Baseline clinical characteristics for the entire population and stratified by high vs. low E/SRe (by the median of 97) are shown in Table 1. Briefly, the mean age was 62 years and 75% were male, 32% had hypertension, 8% had diabetes mellitus, 17% had dyslipidemia, 5% had prior MI, and mean LVEF was 46 ± 9%.

**Table 1:** Baseline characteristics for the entire study population and according to E/SRe

Variable	All	Low E/SRe (< 97)	High E/SRe (> 97)	P-value
	(n = 369)	(n = 185)	(n = 184)	
Age (years)	62 ± 11	59 ± 10	65 ± 12	< 0.001
Male gender, n (%)	277 (75)	140 (76)	137 (75)	0.79
HR (beats per minute)	75 ± 14	72 ± 13	77 ± 15	< 0.001
BMI (kg/m^2^)	27 ± 4	26 ± 4	27 ± 5	0.014
Hypertension, n (%)	118 (32)	44 (24)	74 (40)	< 0.001
Diabetes, n (%)	31 (8)	7 (4)	24 (13)	0.001
Hypercholesterolemia, n (%)	62 (17)	30 (16)	32 (17)	0.76
Current smokers, n (%)	191 (52)	103 (56)	88 (48)	0.13
History of heart failure, n (%)	4 (1)	1 (1)	3 (2)	0.37
Previous MI, n (%)	17 (5)	7 (4)	10 (5)	0.45
Culprit lesion				
Left anterior descending artery, n (%)	178 (48)	75 (41)	103 (56)	0.003
Right coronary artery, n (%)	150 (41)	86 (47)	64 (35)	0.022
Left circumflex coronary artery, n (%)	40 (11)	24 (13)	16 (9)	0.19
Lab work				
Creatinine (µmol/L)	91 [77–108]	90 [77–105]	93 [77–112]	0.20
Peak Troponin I (µg/L)	110 [29–232]	69 [24–188]	163 [39–298]	< 0.001
Echocardiography				
Significant valve disease, n (%)	12 (3)	2 (1)	10 (5)	0.020
Mitral stenosis, n (%)	0 (0)	0 (0)	0 (0)	N/A
Mitral regurgitation, n (%)	7 (2)	1 (0.5)	6 (3)	0.07
Aortic stenosis, n (%)	7 (2)	1 (0.5)	6 (3)	0.07
Aortic regurgitation, n (%)	1 (0.3)	0 (0)	1 (0.5)	0.50
Left ventricular mass index (g/m^2^)	91 [75–111]	86 [71–103]	94 [80–115]	< 0.001
LVEF (%)	46 ± 9	49 ± 8	43 ± 9	< 0.001
LVEDV (mL)	51.2 ± 15.6	49.5 ± 13.2	52.8 ± 17.5	0.041
LVESV (mL)	28.1 ± 11.8	25.7 ± 8.4	30.5 ± 14.1	< 0.001
LVIDd (cm/m2)	2.5 ± 0.3	2.5 ± 0.3	2.6 ± 0.4	0.048
E/A ratio	1.02 [0.83–1.30]	1.04 [0.86–1.31]	1.0 [0.82–1.26]	0.38
e’ (cm/s)	7.4 ± 2.2	8.2 ± 2.1	6.6 ± 2.0	< 0.001
E/e’	10.5 [8.4–12.9]	9.0 [7.5–10.6]	12.3 [10.2–15.0]	< 0.001
MV E velocity (cm/s)	77 ± 19	72 ± 17	81 ± 21	< 0.001
MV A velocity (cm/s)	74 ± 20	69 ± 17	80 ± 22	< 0.001
Left atrial volume (mL/m^2^)	24 [20-29	24 [19-29]	24 [20-28	0.43
Left atrial strain (%)*	30.4 (22.7–40.3)	33.6 (26.8–44.9)	25.9 (20.7–35.2)	< 0.001
Global longitudinal strain (%)	− 12.3 ± 3.7	− 14.3 ± 3.3	− 10.4 ± 2.9	< 0.001
E/SRe	97.2 [78.3–124.7]	78.3 [65.6–87.2]	124.8 [108.2–156.5]	< 0.001

Data are presented as continuous variable showing Gaussian distribution as mean ± standard deviations, numbers (percentage) or median with interquartile range

*BMI* body mass index, *MI* myocardial infarction, *HR* heart rate, E/A ratio; transmitral early filling velocity to filling velocity during atrial contraction, *E/SRe*; ratio of transmitral early filling velocity to global strain rate at early filling phase, *E/e’* ratio of transmitral early filling velocity to early diastolic tissue velocity, *LVEF* left ventricular ejection fraction, *LVEDV* left ventricular end-diastolic volume, *LVESV* left ventricular end-systolic volume, *LVIDd* left ventricular internal dimension at end-diastole

* Avaliable in 299 patients

Patients with high E/SRe had a higher cardiovascular risk profile as they were older, more frequently had hypertension, diabetes, and heart failure. They also more frequently had anterior infarction and larger infarct size. In terms of echocardiographic characteristics, these patients had more extensive LV remodeling as well as more impaired systolic and diastolic function.

During a median follow-up period of 5.6 years (IQR: 5.0–6.1 years), 23 (6%) of the participants developed AF. Baseline characteristics stratified by the endpoint of AF are shown in supplementary table 1. Patients who developed AF were significantly older than those who did not (68 vs 62, p = 0.021). A significantly higher proportion had diabetes in this group (22% vs 8%; p = 0.017), and they presented with impaired diastolic function by the e’ (6.4 vs 7.5 cm/s, p = 0.028) and E/e’ (13.5 vs 10.4, p = 0.001). Those who developed AF exhibited significantly reduced systolic function by GLS (-10.5% vs -12.5%, p = 0.01) but not by LVEF (44 ± 9% vs 46 ± 9%, p = 0.49).

### Predictive value of E/SRe

**Fig. 2:**
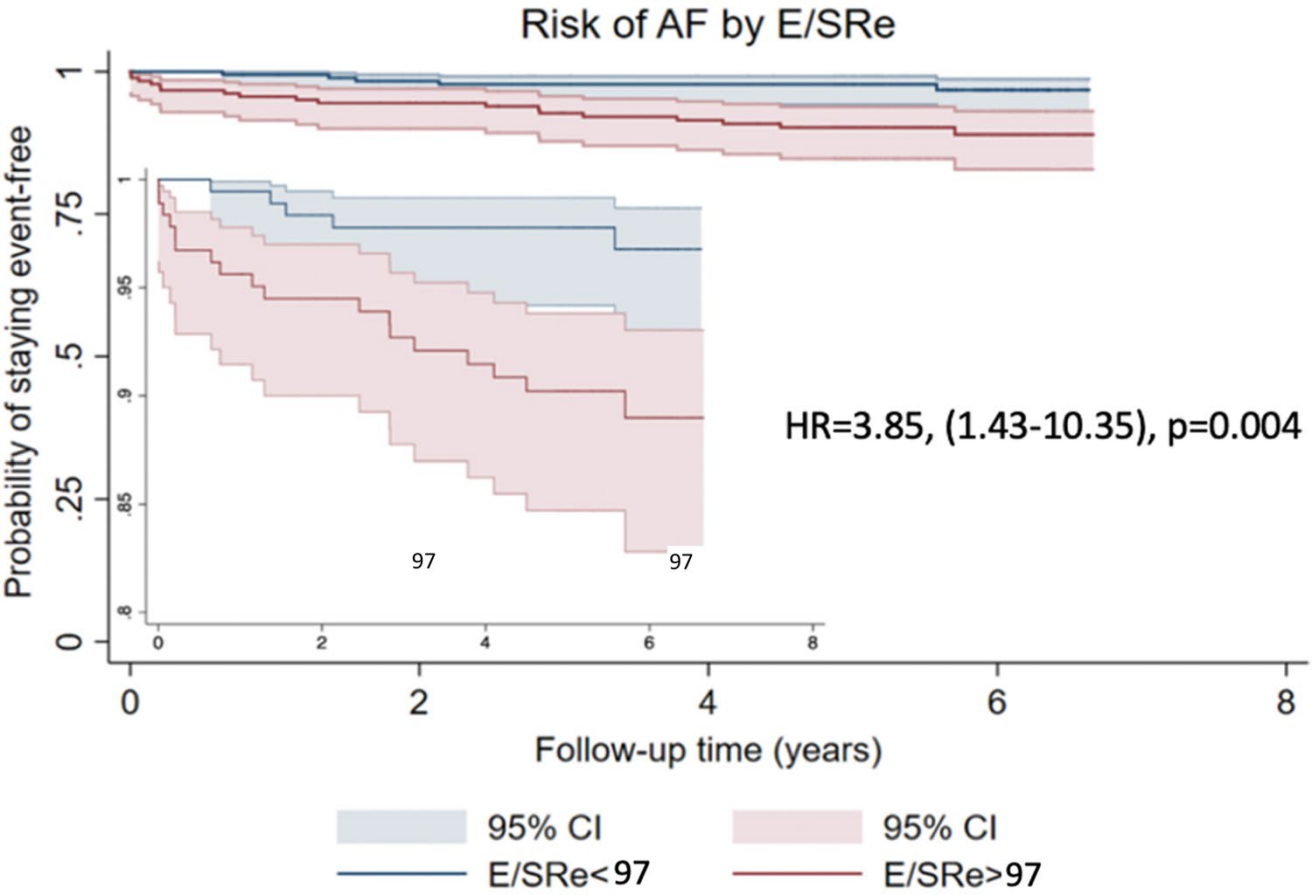
Kaplan Meier curves stratified according to median of E/SRe in patients. Kaplan Meier estimates, showing the risk of AF throughout the study period for patients stratified by high vs. low E/SRe (based on the median value). *E/SRe* ratio of transmitral early filling velocity to early diastolic strain rate, *AF* atrial fibrillation

Compared to patients with low E/SRe, those with high E/SRe had an almost four-fold increased risk of AF during follow-up (HR = 3.85 (1.43–10.35), p = 0.004) (Fig. 2). The incidence rate was significantly higher in patients with high E/SRe as compared to those with low E/SRe (high E/SRe: 20 (95% CI: 13–32) events per 1,000 patient-years; low E/SRe: 5 (95% CI: 2–12) events per 1000 patient-years).

**Fig. 3:**
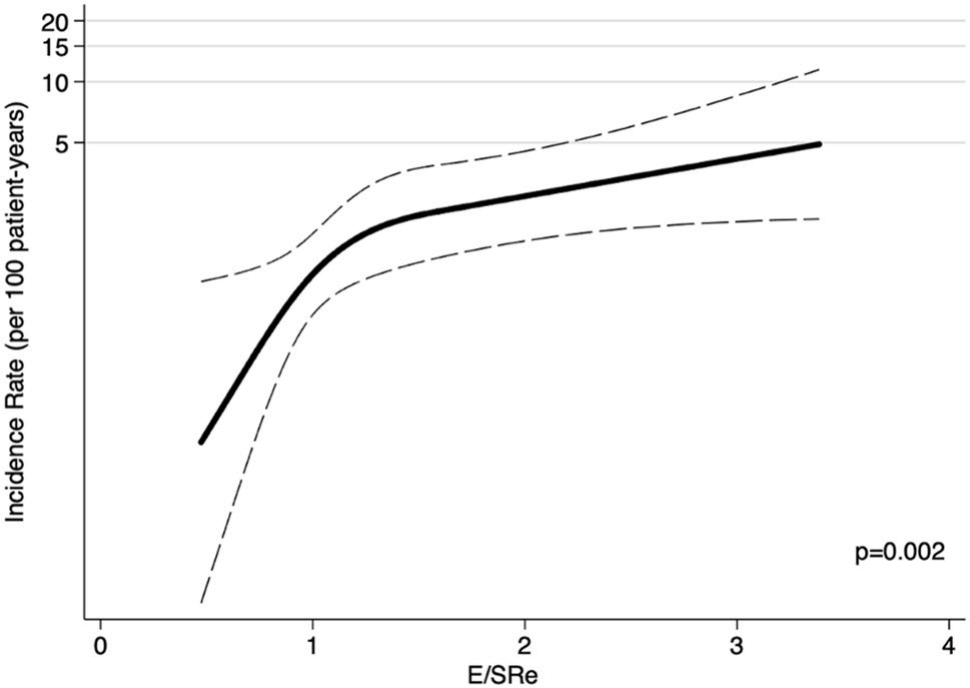
Relationship between E/SRe and outcome. The figure shows a restricted cubic spline curve, illustrating the relationship between E/SRe and the risk of AF. Solid line represents the unadjusted incidence rate and dotted lines represents 95% CI. *CI* confidence

In univariable Cox regression analyses, both E/SRe and E/e’ were significantly associated with AF (E/SRe: HR = 1.06; (1.03–1.10); p < 0.001, per 10 increase; E/e’: HR = 1.11 (1.05–1.17); p < 0.001, per 1 increase). Both measures had a Harrell’s C-statistic of 0.71. Of note, a non-linear association between E/SRe and AF was observed. Showing that an increasing E/SRe was associated with an increased risk of AF as shown in Fig. 3. Furthermore, decreasing LA strain, increasing age, increasing peak TnI, and history of diabetes were also significantly associated with an increased risk of AF (Table 2). However, after multivariable adjustments, E/SRe was the only parameter to remain independently associated with AF (HR: 1.06 (1.00–1.11); p = 0.044, per 10 increase) (Table 2). When applying the same multivariable adjustments for E/e’, no parameter was associated with AF, including E/e’ (HR 1.08 (1.00–1.16), p = 0.06). If we extended the multivariable model to include LA strain (available in a subset, n: 299), E/SRe was still significantly associated with AF (HR 1.09 (1.02–1.16), p = 0.009, per 10 increase), and E/e’ was not (HR: 1.09 (0.98–1.21), p = 0.10, per 1 increase). No effect-modification was observed by sex nor GLS (p for interaction > 0.05) for the association between E/SRe and AF. Moreover, LVEF did not significantly modify the association between E/LVSRe and AF (p for interaction = 0.15). All of the findings were unchanged in multivariable competing risk regression analyses when accounting for all-cause death as a competing event (E/SRe: SHR = 1.05 (1.00–1.11), p = 0.038, per 10 increase; E/e’: SHR = 1.08 (0.98–1.20), p = 0.12, per 1 increase).

**Table 2:** Cox regression with all covariates included in the multivariable models

	HR (95% CI)	p-value
Univariable analysis		
E/SRe, per 10 increase	1.06 (1.03–1.10)	< 0.001
E/e’, per 1 increase	1.11 (1.05–1.17)	< 0.001
Age, per 1 year increase	1.05 (1.01–1.09)	0.012
Male sex	0.59 (0.25–1.40)	0.23
Hypertension	2.07 (0.91–4.70)	0.08
Diabetes	3.11 (1.15–8.38)	0.025
Peak Troponin I, per 100 increase	1.23 (1.03–1.48)	0.023
LVMI, per 10 g/m^2^ increase	1.06 (0.94–1.21)	0.35
LVEF, per 1% decrease	1.02 (0.98–1.07)	0.38
Heart rate, per 1 bpm increase	1.02 (1.00–1.05)	0.10
Significant valve disease	1.45 (0.19–10.73)	0.72
LAD lesion	1.40 (0.61–3.20)	0.42
LA strain*, per 1% decrease	1.07 (1.02–1.12)	0.005
Multivariable analysis		
E/Sre, per 10 increase	1.06 (1.00–1.11)	0.044
Age, per 1 year increase	1.04 (1.00–1.11)	0.07
Male sex	0.81 (0.31–2.15)	0.68
Hypertension	1.24 (0.51–2.99)	0.63
Diabetes	2.24 (0.78–6.42)	0.14
Peak Troponin I, per 100 increase	1.17 (0.95–1.44)	0.15
LVMI, per 10 g/m^2^ increase	1.01 (0.88–1.17)	0.84
LVEF, per 1% decrease	0.98 (0.93–1.03)	0.45
Heart rate, per 1 bpm increase	1.01 (0.98–1.05)	0.37
Significant valve disease	1.13 (0.13–9.56)	0.91
LAD lesion	1.13 (0.47–2.74)	0.78

*LVMI* left ventricular mass index, *LVEF* left ventricular ejection fraction, *LAD*: left anterior descending, *LA*: left atrial, *BPM* beats per minute

*available in 299 patients

E/SRe was also significantly associated with AF in patients with normal filling pressure (E/e’ < 14) (n = 301) in unadjusted analysis (HR = 1.09 (1.01–1.17); p = 0.030, per 10 increase). After multivariable adjustments E/SRe remained associated with AF (HR = 1.12 (1.00–1.26); p = 0.048, per 10 increase).

When looking at well-established echocardiographic predictors of AF, abnormal E/e (> 14) and TR gradient (> 2.8 m/s), were significantly associated with an increased risk of AF, whereas abnormal LVEF, GLS and LAVi were not (Supplementary table 2). However, on a continuous scale, only E/SRe remained significantly associated with AF after multivariable adjustments as described previously.

## Discussion

This study is currently the largest to investigate the value of E/SRe for predicting AF in patients with STEMI treated with pPCI. We demonstrated that patients with high E/SRe had more extensive LV remodeling as well as more impaired systolic and diastolic function compared to those with low E/SRe. Secondly, E/SRe remained a consistent predictor of AF in both multivariable models as well as in competing risk analyses. E/SRe was capable of predicting AF in patients with normal filling pressure as estimated by E/e’ (E/e’ < 14).

### Estimating LV filling pressure

As previously mentioned, AF is the most common cardiac arrhythmia following AMI. Several factors increase the risk of developing AF after AMI, particularly elevated LV filling pressure associated with diastolic dysfunction. As LV filling pressure increases—as often observed after AMI–an increased afterload pressure gradient develops across the LV and LA chambers, causing LA remodeling, and therefore increases the risk of developing AF [24]. E/e’ has long been used to assess LV filling pressure in a non-invasive approach and is one of the key guideline-based methods for estimating filling pressure [19, 25]. However, E/e’ is subjected to several technical limitations (as outlined below), and other markers have therefore been proposed, including E/SRe. Several studies have demonstrated an association between invasively measured LV end diastolic pressure and E/SRe [14].

The increasing usage of two-dimensional myocardial speckle tracking, which is a fully automatic technique, along with its implementation into clinical guidelines, strain rate has a potential to play a key role in echocardiographic assessments in the future. E/SRe is therefore a fast, non-invasive, and accurate way of estimating the LV filling pressure in patients undergoing echocardiography. Recently, the measure has been benchmarked even further for clinical use as normal values have been defined based on 1623 healthy individuals from the Copenhagen City Heart study, with an overall upper reference limit of normality for E/SRe of 80 [26].

### Prognostic value of E/SRe compared with E/e’

In our study, we found that both E/SRe and E/e’ were significantly associated with AF and had similar predictive value by Harrell’s C-statistics. However, after adjusting for confounders and further accounting for all-cause death and AF as a competing event, only E/SRe remained associated with the outcome. In addition to this, several technical circumstances favor the use of E/SRe. It is well-established that E/e’ often lacks accuracy due to several technical limitations that challenge its interpretation, including susceptibility to sampling location, myocardial tethering and significant errors with angulation > 20 degrees [27]. In contrast, strain rate derived from speckle-tracking is not angle dependent nor is it affected by myocardial tethering [20]. Furthermore, it may better reflect the LV relaxation as it is a global measurement taking the entire LV myocardium into account, in contrast to e’ witch only measures velocities at the basal segments near the mitral annulus of the LV. Furthermore, regional wall motion abnormalities- which is frequently observed in MI—near the sampling region along with mitral annular calcifications, may underestimate values of e’ and thereby falsely classify patients as having diastolic dysfunction despite overall normal LV relaxation. Consequently, E/SRe may facilitate diastolic function assessment when E/e’ is technically unreliable. However, our findings suggest that its value is not restricted to these settings since we also observed that E/SRe was a predictor of AF in patients without elevated LV filling pressure by E/e’. By conventional standards these patients may have either been classified as having normal diastolic function or indeterminate diastolic function, and E/SRe could help properly classify and risk stratify these patients.

### Related studies and clinical relevance

One former small study conducted by Bonapace et al. have investigated the relationship between E/SRe and the development of new-onset AF. They investigated a cohort of 180 type-2 diabetic patients and found an increase in E/SRe in those who developed AF, similarly to our findings. However, E/SRe was not significantly associated with AF in the multivariable logistic regression. However, they had a low number of events (14 events) and their findings may therefore have been influenced by low power [16]. While there is sparse data regarding the association between E/SRe and AF, the prognostic value of E/SRe has been investigated in multiple other settings. Ersbøll et al. showed in a cohort of AMI patients (n = 1100), that E/SRe had a better prognostic value than E/e’ and was a stronger predictor of a composite endpoint HF, AF, stroke, and all-cause mortality. Remarkably there was no association between E/SRe and AF, due to the fact that this study was driven by HF and all cause death [15] Lassen et al. have further demonstrated E/SRe as an important and reliable prognostic measurement superior to E/e’ in a number of studies in specific patient populations including type 2 diabetes, heart failure with reduced ejection fraction, acute coronary syndrome, and the overall general population in relation to outcomes including AMI, HF, AF, and mortality [21–23, 28]. Similar to our findings, these studies also showed a non-linear relationship between E/SRe and outcome. Lassen et al. [21, 22] further found an effect modification with systolic function such that E/SRe was a particularly strong predictor of outcome in patients with preserved systolic function. In our study, we did not observe any effect-modification, which could reflect the relatively few numbers of events.

### LA volume, strain and LV filling pressure

The LA is directly exposed to pressure from the LV during diastole and will therefore enlarge as LV filling pressure increases. Accordingly, elevated LV filling pressure and abnormal LA volume is associated with AF [24]. No difference in LA volume by outcome was observed in our population, which may be because LA remodeling develops as a consequence of chronic pressure overload, and therefore remodeling of the LA may not appear in the acute settings of MI within the brief time period from infarct to echocardiography (median of 2 days in this study).

Besides E/SRe and LA volume, LA strain has proven valuable. A recent study found that LA strain did not add additional prognostic value, when both LA volume and LV filling pressure was already obtained [29]. Intrinsic LA remodeling and dysfunction may be a key factor in the pathogenesis of AF. In our study, however, we found that E/SRe remained an independent predictor of outcome even after adjusting for LA strain, emphasizing that the association is not driven exclusively by changes in the LA. Rather, LV abnormalities may be the first change of clinical significance that eventually leads to LA functional impairment. Our findings further indicate that employing E/SRe in the diastolic dysfunction assessment was useful for identifying patients at-risk of outcome, who would not otherwise be perceived as having elevated filling pressure. The results emphasize that E/SRe could be a useful diastolic measure to be used in concert with conventional measures of diastolic function.

Although strain rate has shown potential in risk stratification across several studies, the technique itself has its limitations. The technique relies–to a larger extent than GLS–on adequate temporal resolution, since a low frame rate may lead to erroneous sampling of peak amplitudes [30]. Consequently, operators should keep this technical aspect in mind. This issue may be resolved with future technical developments as high-frame rate applications become available [31]. Our results should be interpreted in light of this technical limitation, which also emphasizes that our findings are hypothesis-generating and should be validated externally and further validated once high-frame rate techniques become widely available.

## Limitations

This study has certain limitations. While strain rate measures could offer valuable insights with respect to cardiac function, these measures are dependent on a high temporal resolution, which may not be achievable with conventional speckle tracking echocardiography. Even with a relatively high frame rate of 86 (IQR: 76 to 102) fps in this study, this implies that our findings will need to be validated externally, preferably with high frame rate software solutions. The number of clinical events was relatively few (23 events) compared to the number of variables included in our multivariable models, which could introduce an issue of overfitting. However, this method holds merit, as long as the focus is to account for confounding variables to test the strength of the association [32]. As the diagnosis of AF was obtained from nationwide registries, no systematic rhythm monitoring was applied, and consequently, we do not have information as to how AF was diagnosed, which runs a risk of missing potential AF events. However, the Danish National Patient Registry has previously been found to be accurate with respect to AF diagnosis [27]. Some parameters were not collected in this study, including NT-proBNP and antiarrhythmic medication. Consequently, our study may therefore be subjected to uncorrected confounding. The number of clinical events was relatively few (23 events) which relies on the intermediate follow-up period of 5.6 years. Consequently, studies detailing the association between E/SRe and the long-term risk of AF are still warranted.

## Conclusion

E/SRe is an independent predictor of AF after STEMI treated with pPCI, even in subjects with seemingly normal filling pressure. High E/SRe is associated with a nearly four-fold increased risk of AF. Accordingly, this measure could assist the risk stratification process for AF after AMI. As the current literature of E/SRe is limited, it is necessary to validate these findings in larger studies which also includes a systematic rhythm monitoring approach for detecting AF.

### Supplementary Information

Below is the link to the electronic supplementary material.

Supplementary file1 (DOCX 25 kb)
