# Supplementary material for: Ratio of early transmitral inflow velocity to early diastolic strain rate predicts atrial fibrillation following acute myocardial infarction
Source: Int J Cardiovasc Imaging. 2023 Nov 13;40(2):331–40. doi: 10.1007/s10554-023-02991-y (PMC10884066; doi:10.1007/s10554-023-02991-y)
Supplement: Supplementary file 1 — Supplementary file1 (DOCX 25 kb) [file 10554_2023_2991_MOESM1_ESM.docx]

SUPPLEMENTARY MATERIAL

Ratio of Early Transmitral Inflow Velocity to Early Diastolic Strain Rate Predicts Atrial Fibrillation following Acute Myocardial Infarction

Caroline Løkke Bjerregaard, MB1; Flemming Javier Olsen, MD1,2;
Mats Christian Højbjerg Lassen, MD1; Anne-Sophie Winther Svartstein, MB^1^; Thomas-Fritz Hansen, MD1; Søren Galatius, MD, DMSc3; Allan Iversen, MD, PhD1,2; Sune Pedersen, MD, PhD1; Tor Biering-Sørensen, MD, PhD, MPH1,2

1 Department of Cardiology, Copenhagen University Hospital - Herlev and Gentofte, Copenhagen, Denmark.
2 Department of Biomedical Sciences, Faculty of Health and Medical Sciences, University of Copenhagen, Copenhagen, Denmark
3 Department of Cardiology, Copenhagen University Hospital – Bispebjerg and Frederiksberg, Copenhagen, Denmark

Running title: Diastolic strain rate predicts AF

**Correspondence:**

Caroline Løkke Bjerregaard
Cardiovascular Non-Invasive Imaging Research Laboratory, Department of Cardiology, Herlev & Gentofte Hospital, University of Copenhagen, Denmark
Niels Andersens Vej 65, 2900 Hellerup, Denmark
Phone: +45 50 45 86 18 ; Fax: +45 39 77 73 81
E-mail: bjerregaard.caroline@gmail.com

**Supplementary table**

**Table 1**

Baseline characteristics stratified by the endpoint of AF

| Variable | Non-AF | AF | P-value |
| --- | --- | --- | --- |
|  | (n=346) | (n=23) |  |
| Age (years) | 62 ± 11 | 68 ± 12 | 0.021 |
| Male gender, n (%) | 252 (76) | 15 (65) | 0.26 |
| HR (beats per minute) | 74 ± 14 | 79 ± 19 | 0.14 |
| BMI (kg/m^2^) | 27 ± 4 | 27 ± 4 | 0.48 |
| Hypertension, n (%) | 107 (31) | 11 (48) | 0.09 |
| Diabetes, n (%) | 26 (8) | 5 (22) | 0.017 |
| Hypercholesterolemia, n (%) | 56 (16) | 6 (26) | 0.22 |
| Current smokers, n (%) | 182 (53) | 9 (39) | 0.21 |
| History heart failure, n (%) | 4 (1) | 0 (0) | 1.00 |
| Previous MI, n (%) | 17 (5) | 0 (0) | 0.61 |
| **Culprit lesion** |  |  |  |
| Left anterior descending artery, n (%) | 165 (48) | 13 (57) | 0.41 |
| Right coronary artery, n (%) | 141 (41) | 9 (39) | 0.88 |
| Left circumflex coronary artery, n (%) | 39 (11) | 1 (4) | 0.30 |
| **Lab work** |  |  |  |
| Creatinine (µmol/L) | 91 [77-108] | 91 [79-116] | 0.56 |
| Peak Troponin I (µg/L) | 100 [28-230] | 177 [40-349] | 0.19 |
| **Valve disease** |  |  |  |
| Valve disease, n (%) | 11 (3) | 1 (4) | 0.54 |
| Mitral stenosis, n (%) | 0 (0) | 0 (0) | N/A |
| Mitral regurgitation, n (%) | 6 (2) | 1 (4) | 0.37 |
| Aortic stenosis, n (%) | 7 (2) | 0 (0) | 1.00 |
| Aortic regurgitation, n (%) | 1 (0.3) | 0 (0) | 1.00 |
| **Echocardiography** |  |  |  |
| Left ventricular mass index (g/m^2^) | 92 [75-111] | 85 [78-126] | 0.83 |
| LVEF (%) | 46 ± 9 | 44 ± 9 | 0.47 |
| E/A ratio | 1.02 [0.83–1.30] | 1.02 [0.81-1.38] | 0.92 |
| e’ (cm/s) | 7.5 ± 2.2 | 6.4 ± 2.0 | 0.028 |
| E/e’ | 10.4 [8.2-12.6] | 13.5 [9.7-15.6] | 0.001 |
| MV E velocity (cm/s) | 76 ± 19 | 81 ± 19 | 0.24 |
| MV A velocity (cm/s) | 74 ± 20 | 76 ± 24 | 0.74 |
| Left atrial volume (mL/m^2^) | 24 [20-28] | 25 [19-30] | 0.53 |
| Left atrial strain (%)* | 30.9 (23.4-40.9) | 21.4 (15.4-26.4) | 0.002 |
| Global longitudinal strain (%) | -12.5 ± 3.6 | -10.5 ± 4.0 | 0.010 |
| E/SRe | 95.8 [75.1-122.7] | 129.7 [97.8-171.7] | 0.001 |

- Available in 299 patients

Data are presented as continuous variable showing Gaussian distribution as mean ± standard deviations, numbers (percentage) or median with interquartile range
BMI: body mass index; MI: myocardial infarction, HR; heart rate, E/A ratio; transmitral early filling velocity to filling velocity during atrial contraction, E/SRe; global strain rate at early filling phase, E/e’; ratio of transmitral early filling velocity to early diastolic tissue velocity, LVEF; left ventricular ejection fraction

**Supplementary Table 2**

Echocardiographic abnormalities and AF

|  | HR (95% CI) | P-value |
| --- | --- | --- |
| Univariable model |  |  |
| E/SRe > 97 | 3.85 (1.43 – 10.36) | 0.008 |
| LAVI > 34 mL/m^2^ | 1.34 (0.40 - 4.51) | 0.64 |
| GLS < 16% | 2.26 (0.53 - 9.63) | 0.27 |
| E/e’ > 14 | 4.42 (1.96 – 10.06) | 0.001 |
| TR > 2.8 m/s* | 3.33 (1.12 – 9.93) | 0.031 |
| LVEF < 50% | 1.49 (0.56 – 3.79) | 0.40 |

E/SRe; ratio of transmitral early filling velocity to global strain rate at early filling phase, LAVI; Left atrial volume index, GLS; Global longitudinal strain, E/e’; ratio of transmitral early filling velocity to early diastolic tissue velocity, TR; Tricuspid regurgitation, LVEF; Left ventricular ejection fraction.

* available in 259 patients
